# Metagenomic analysis reveals that modern microbialites and polar microbial mats have similar taxonomic and functional potential

**DOI:** 10.3389/fmicb.2015.00966

**Published:** 2015-09-23

**Authors:** Richard Allen White, Ian M. Power, Gregory M. Dipple, Gordon Southam, Curtis A. Suttle

**Affiliations:** ^1^Department of Microbiology and Immunology, University of British ColumbiaVancouver, BC, Canada; ^2^Department of Earth, Ocean and Atmospheric Sciences, University of British ColumbiaVancouver, BC, Canada; ^3^School of Earth Sciences, University of QueenslandBrisbane, QLD, Australia; ^4^Department of Botany, University of British ColumbiaVancouver, BC, Canada; ^5^Canadian Institute for Advanced ResearchToronto, ON, Canada

**Keywords:** microbialites, non-lithifying microbial mats, metagenomic assembly, carbon sequestration, Gemmatimonadetes, cyanobacteria

## Abstract

Within the subarctic climate of Clinton Creek, Yukon, Canada, lies an abandoned and flooded open-pit asbestos mine that harbors rapidly growing microbialites. To understand their formation we completed a metagenomic community profile of the microbialites and their surrounding sediments. Assembled metagenomic data revealed that bacteria within the phylum Proteobacteria numerically dominated this system, although the relative abundances of taxa within the phylum varied among environments. Bacteria belonging to Alphaproteobacteria and Gammaproteobacteria were dominant in the microbialites and sediments, respectively. The microbialites were also home to many other groups associated with microbialite formation including filamentous cyanobacteria and dissimilatory sulfate-reducing Deltaproteobacteria, consistent with the idea of a shared global microbialite microbiome. Other members were present that are typically not associated with microbialites including Gemmatimonadetes and iron-oxidizing Betaproteobacteria, which participate in carbon metabolism and iron cycling. Compared to the sediments, the microbialite microbiome has significantly more genes associated with photosynthetic processes (e.g., photosystem II reaction centers, carotenoid, and chlorophyll biosynthesis) and carbon fixation (e.g., CO dehydrogenase). The Clinton Creek microbialite communities had strikingly similar functional potentials to non-lithifying microbial mats from the Canadian High Arctic and Antarctica, but are functionally distinct, from non-lithifying mats or biofilms from Yellowstone. Clinton Creek microbialites also share metabolic genes (*R*^2^ < 0.750) with freshwater microbial mats from Cuatro Ciénegas, Mexico, but are more similar to polar Arctic mats (*R*^2^ > 0.900). These metagenomic profiles from an anthropogenic microbialite-forming ecosystem provide context to microbialite formation on a human-relevant timescale.

## Introduction

Modern microbialites provide an analog for understanding the structure, composition and function of early microbial communities dating back dating back 3.5 Gya (Grotzinger and Knoll, 1999; Dupraz and Visscher, 2005; Schopf, 2006). Microbialites are microbially lithified organosedimentary structures that manifest the carbonate macrofabric as stromatolites (from the Greek *stromat*; bed-covering, *lithos*; stone, as a layered internal structure with laminated fabrics), and thrombolites (from the Greek *thrombos*, clot; *lithos*, stone as a non-layered, non-laminated structure with clotted fabrics) (Burne and Moore, 1987; Perry et al., 2007; Riding, 2011). Modern microbialites are mainly constructed from calcium carbonate, either as calcite or aragonite), but in rare cases can occur as magnesium carbonate (e.g., hydromagnesite) (Couradeau et al., 2011). Microbialites are globally distributed in diverse environments including marine (Reid et al., 2000; Burns et al., 2004), freshwater (Ferris et al., 1997; Laval et al., 2000; Gischler et al., 2008), hypersaline (Allen et al., 2009; Goh et al., 2009), hot springs (Bosak et al., 2012), and even terrestrial environments, such as landfills (Maliva et al., 2000) and caves (Lundberg and McFarlane, 2011).

Biologically-induced mineralization involves the microbial alteration of water chemistry causing mineral saturation and precipitation (Dupraz et al., 2009). Microbial processes that cycle carbon, particularly within microenvironments, are important for inducing carbonate precipitation under appropriate chemical conditions (e.g., alkaline pH and sufficient cations; Spanos and Koutsoukos, 1998; Dupraz et al., 2009). For instance, cyanobacteria can cause alkalinization through photosynthesis, thereby driving pH to more alkaline conditions that favor carbonate precipitation (Thompson and Ferris, 1990). (Equation 1) ${\text{HCO}}_{3}^{-}+{\text{H}}_{2}\text{O}+hv\to {\text{CH}}_{2}\text{O}+{\text{OH}}^{-}+{\text{O}}_{2}↑$. Microbial cell walls and exopolymeric substances (EPS) may provide surfaces for mineral nucleation and aid in concentrating cations (e.g., Ca^2+^) due to adsorption by negatively charged functional groups (e.g., R-COO^−^) (Schultze-Lam et al., 1996). Additionally, heterotrophic bacteria can increase the availability of dissolved inorganic carbon (DIC) for carbonate precipitation through the degradation of organics (Von Knorre and Krumbein, 2000). (Equation 2) $2{\text{CHO}}_{2}^{-}+{\text{O}}_{2}\to 2{\text{HCO}}_{3}^{-}$. Although aragonite is supersaturated in the Clinton Creek open-pit pond, studies of non-marine environments exhibiting calcifying cyanobacteria show that a 9.5 to 15-fold supersaturation with respect to calcite is required for precipitation to occur (Arp et al., 2001). Such biological activity, especially in microenvironments where carbonate precipitation may be occurring, may increase pH, and/or increase cation and DIC concentrations.

Microbial processes that cycle carbon may also induce carbonate precipitation under certain geochemical conditions (e.g., alkaline pH and sufficient cations; Dupraz et al., 2009). Photosynthesis by cyanobacteria may result in the alkalization of microenvironments by producing hydroxyl anions, causing an increase in pH (Thompson and Ferris, 1990; Ludwig et al., 2005; Tesson et al., 2008); whereas, degradation of organics may increase the concentration of DIC (Slaughter and Hill, 1991; Van Lith et al., 2003).

**Figure 1:**
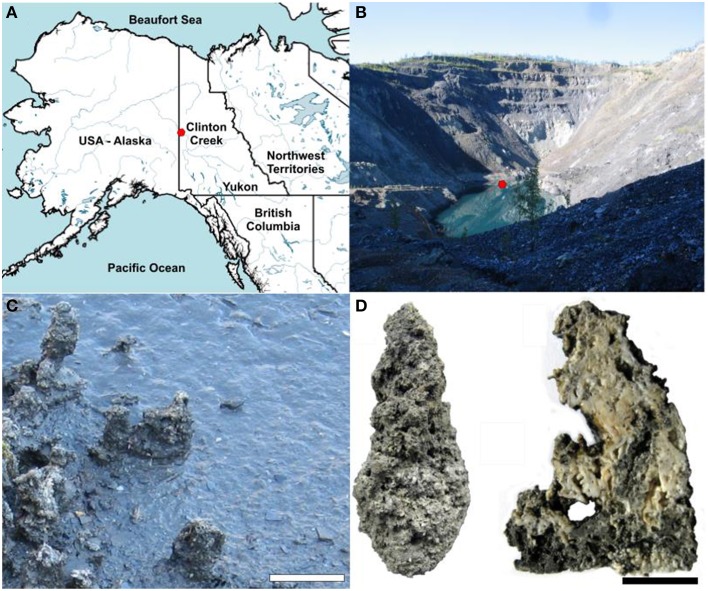
**Clinton Creek sample site and examples of microbialite morphology. (A)** Map of northwestern North America illustrating the location of Clinton Creek Yukon, Canada. **(B)** Photograph of the Clinton Creek open pit pond, the red dot indicating the sampling location. **(C)** Photograph of the microbialites along the periphery of the open pit pond. Scale bar equals 15 cm. **(D)** Complete microbialite and cross-section of a microbialite. Scale bar represents 5 cm.

In the present study we examined the microbial communities associated with microbialites found in a flooded and abandoned open-pit asbestos mine (64°26′42″N, 140°43′25″W) referred to as Clinton Creek, and located in the subarctic, ~77 km northwest of Dawson City, Yukon, Canada (Figure 1), and which was previously studied to elucidate the geology of asbestos deposits (Htoon, 1979) and for its potential for sequestering carbon dioxide in mine wastes (Wilson et al., 2009). The microbialites at Clinton Creek are unusual in that they have estimated accretion rates of up to ~5 mm per year (Power et al., 2011a), much higher than other modern microbialite-forming systems including Highbourne Cay (~0.33 mm per year) (Planavsky and Ginsburg, 2009), Shark Bay (0.4 mm per year) (Chivas et al., 1990), and Pavilion Lake (0.05 mm per year) (Brady et al., 2009). Consequently, the Clinton Creek microbialites should be excellent models for understanding the biological processes responsible for microbialites formation.

Studies have examined the diversity of microbialites using both metagenomic and 16S rDNA sequencing. Metagenomic studies have focused mainly on marine systems (Reid et al., 2000; Burns et al., 2004; Papineau et al., 2005; Allen et al., 2009; Goh et al., 2009; Khodadad and Foster, 2012; Mobberley et al., 2013), with Cuatro Ciénegas being the only reported metagenomic investigation of freshwater microbialites (Breitbart et al., 2009). In contrast, 16S rDNA sequencing has been used to examine the diversity of freshwater microbialites in Lake Van (López-García et al., 2005), Pavilion Lake (Russell et al., 2014), Ruidera Pools (Santos et al., 2010), Lake Alchichica (Couradeau et al., 2011), and Cuatro Ciénegas (Centeno et al., 2012). The extent to which microbialite communities are similar or different from those in surrounding sediments and waters remains an unresolved but important question. The microbial communities in the surrounding sediments provide the environmental context needed to better constrain common and unique aspects of microbialite community structure and function. This information may be used to uncover conserved patterns of microbial community assembly and the metabolic pathways mediating microbialite formation under different environmental conditions.

In the present study, we use a metagenomic approach to explore the structure and function of Clinton Creek microbialites in relation to adjacent sediments in order to examine the metabolic drivers of microbialite growth in this freshwater system. We focus on metabolic pathways mediating photosynthetic or heterotrophic carbonate precipitation and the taxonomic distribution of these pathways. We then compare the metagenomic data from Clinton Creek microbialites and sediments to diverse non-lithifying mats, sediments, and microbialites to better define the conserved microbialite community structure and function.

## Materials and methods

### Site description and water chemistry

The conditions at the Clinton Creek mine are highly conducive to microbialite formation and are described extensively in Power et al. (2011a), and are summarized briefly below. The photic zone likely occupies the full depth of the open pit pond and there is minimal nutrient input due to the lack of surrounding soil. Sediments are composed of chrysotile, quartz, muscovite, kaolinite, as well as minor amounts of aragonite and trace calcite. The microbialites are columnar, up to 15 cm in height, and are primarily composed of aragonite with spherulitic fabric (Figure 1D). The open pit water is subsaline (Na^+^ 17.6–35.7 mg L^−1^ and K^+^ 2.7–5.2 mg L^−1^), oligotrophic (undetectable phosphate), alkaline in pH (8.4), possessing a cation concentrations distribution of Mg^2+^ >> Ca^2+^ >> Na^+^ > K^+^ > Si^4+^, while anions concentrations were ${\text{SO}}_{4}^{2-}$ >> DIC > Cl^−^ (Table 1). As is common in microbialite forming systems (Dupraz et al., 2009; Lim et al., 2009), the water is oligotrophic with very low iron concentrations and undetectable phosphate which is common in microbialite forming systems (Dupraz et al., 2009; Lim et al., 2009). The water is supersaturated with respect to aragonite (saturation index = 0.6), the dominant mineral forming the microbialites, as well as calcite [CaCO_3_].

**Table 1:** **Clinton Creek water chemistry**.

**Year**	**pH**	**Alkalinity (mg ${\text{HCO}}_{3}^{-}$L^−1^)**	**δ^13^C – DIC ‰ VPDB**	**Cations (mg L^−1^)**							**Anions (mg L^−1^)**			
				**Mg^2+^**	**Ca^2+^**	**Si^4+^**	**Na^+^**	**K^+^**	**Fe^3+^**	**Al^3+^**	**Cl^−^**	**${\text{SO}}_{4}^{2-}$**	**${\text{NO}}_{3}^{-}$**	**${\text{PO}}_{4}^{3-}$**
2004	8.42	177	–	356.1	111.9	0.8	23.9	2.7	0.0	0.0	30.8	1315	–	–
2005	8.36	243	–	350.0	95.7	0.7	35.7	5.2	0.0	0.0	35.6	2378	0.52	–
2007	8.40	231	−7.8	519.7	118.8	–	30.8	5.2	–	–	33.2	1997	–	–
2011	7.90	190	−13.03	276.8	61.6	2.1	17.6	2.7	0.03	0.2	17.5	1600	1.5	–

### Sampling, DNA extraction, purity, and concentration measurements

Microbialites and sediment samples were obtained in July 2011. Triplicate sediment and microbialite samples were taken ~10 m apart. Microbialites were ground with mortar and pestle under liquid nitrogen prior to DNA extraction. Community genomic DNA was extracted from triplicate 10 g microbialite and sediment subsamples using a PowerMax soil DNA isolation kit (MoBio Laboratories, Inc., Carlsbad, CA, USA), following the manufacturer's instructions. DNA concentrations were determined using a Nanodrop-3300 (ThermoFisher, Nandrop Wilmington, DE) with PicoGreen® reagent according to the manufacturer's instructions (Invitrogen, Carlsbad, CA). Purity of extracted DNA and samples was determined by absorbance (260/280 and 260/230 ratios) using a Nanodrop-1000 (ThermoFisher, Nandrop Wilmington, DE). Genomic DNA from each replicate was pooled prior to Illumina library construction.

### Illumina Hiseq/Miseq library construction quality control and quantification

For Illumina library construction, DNA was sheared by ultrasonication (Covaris M220 series, Woburn, MA), and the fragments end-paired, A-tailed (Lucigen NxSeq DNA prep kit, Middleton, WI), and ligated to TruSeq adapters (IDT, Coralville, Iowa); small fragments were removed twice using magnetic beads (Beckman Coulter, Danvers, MA) (White III et al., 2013a,b; White III and Suttle, 2013). No PCR enrichment was used to amplify libraries to avoid PCR duplication bias. Libraries were checked for size and adapter-dimers using a Bioanalyzer HighSens DNAchip (Agilent). Libraries were quantified using Qubit (Invitrogen, Carlsbad, CA), according to the manufacturer's instructions, by qPCR using a microfluidic digital PCR quantified standard curve (White III et al., 2009). The resulting libraries were pooled, and sequenced using both 250 and 100 bp paired-end sequencing on the MiSeq (GenoSeq UCLA Los Angeles, CA) and HiSeq (McGill University/Génome Québec, Montreal, QC) platforms, respectively.

### Analysis of illumina sequencing data

Raw Illumina data was screened for PhiX spike-in contaminants sequencing data using Bowtie2 then removed using Picard tools (White III et al., 2013a,b; White III and Suttle, 2013). Reads were quality checked using FastQC, then paired-end reads merged by FLASH and assembled with the Ray assembler (kmer size: 39 and 55) (Boisvert et al., 2010, 2012; White III et al., 2013a,b; White III and Suttle, 2013). The assemblies were selected based on the number of contigs (>100 bp), N50/N90 values, longest contig, and total length (bp) of the assembly (Table 2). Based on these analyzes, a kmer size 39 was used for all further analysis (Table 2). A kmer size of 55 generally yielded longer but fewer contigs, which would not allow for a comparable differential analysis between microbialites and sediments (Table 2). Only contigs with >2x read coverage were used in analysis with average coverage of 3x for both the microbialite and sediment contigs. Nevertheless, only 0.64 and 1.74% of the raw reads from the sediment and microbialite metagenomes, respectively, assembled into contigs, indicating that both environments had complex microbial communities. FragGeneScan was used to predict and translate contig open reading frames (ORFs) (Rho et al., 2010) and ProPas (Wu and Zhu, 2012) was used to calculate predicted protein isoelectric points (pI).

**Table 2:** **Assembly statistics for Clinton Creek metagenomes**.

	**Microbialite (*k* = *39*)**	**Microbialite (*k* = *55*)**	**Sediment (*k* = *39*)**	**Sediment (*k* = *55*)**
No. Contigs >100 bp	109,722	689	59,928	171
Total length (Bp) >100bp	27,180,461	219,687	12,690,391	151,415
Mean >100 bp	247	318	211	885
N50 >100 bp	248	289	200	1307
N90 >100 bp	184	231	160	383
Med Length >100 bp	240	269	186	582
No. Contigs >500 bp	371	46	557	99
Total length (Bp) >500 bp	230,702	37,861	508,559	127,068
Mean >500 bp	621	823	913	1283
N50 >500 bp	588	611	849	1591
Med >500 bp	574	566	608	916
Largest	9491	9752	33,503	5896
Total GC count	16,801,673	135,459	7,329,632	82,490
GC%	61.82	61.66	57.76	54.48

k = kmer size (bp).

The assemblies were annotated using Metagenomic Rapid Annotations using Subsystems Technology (MG-RAST) (Meyer et al., 2008). MG-RAST analysis of the contigs, used BLAT (BLAST-like Alignment Tool) annotations based on hierarchical classification against M5RNA (MG-RAST ribosomal specific database), SEED subsystems and RefSeq databases with a minimum *e*-value cutoff of 10^−5^, a minimum percent identity cutoff of 60%, and a minimum alignment length cutoff of 15 base pairs. The SEED, RefSeq and M5RNA (MG-RAST rRNA database) classifications were normalized using relative count abundances for each sample. Principal component analysis (PCA) for the normalized RefSeq classifications (top 25) used R (version 3.0.3) libraries Ecodist (dissimilarity-based functions for ecological analysis), and pvclust (hierarchical clustering with *p*-values via multiscale bootstrap resampling) using ward clustering and Bray-Curtis distance metric at a thousand replicates (Suzuki and Shimodaira, 2006). PCA for the normalized RefSeq classifications was plotted using R library ggplot2 (Wickham, 2009). A dotplot of the normalized RefSeq classifications (top 25), was completed using R libraries Reshape2, using the melt function, then plotted using ggplot2 (Wickham, 2009). The annotations were parsed by custom python scripts and analyzed using statistical analysis of metagenomic profiles (STAMP) (Parks and Beiko, 2010). MG-RAST annotations using SEED subsystems for Clinton Creek microbialite and sediment contigs were loaded into STAMP and compared for metabolic potential using a one sided G-test (w/Yates' + Fisher's), alternative to the chi-squared, with asymptomatic confidence intervals (0.95) using Benjamini-Hochberg FDR procedure (Parks and Beiko, 2010).

In addition to MG-RAST, metabolic pathways were predicted using MetaPathways, a modular pipeline for gene prediction and annotation that uses pathway tools and the MetaCyc database to construct environmental pathway/genome databases (ePGBDs) (Konwar et al., 2013). Metapathways uses the seed-and-extend homology search algorithm LAST (local alignment search tool) for annotations of ORFs with a minimum of 180 bp and minimum alignment length cutoff of 50 (Kiełbasa et al., 2011). Venn diagrams were constructed from predicted MetaCyc pathways based on normalized pathway size and the number of ORFs associated with each pathway using R libraries then plotted using ggplot2 (Wickham, 2009).

### Comparative metagenomics

MG-RAST annotations for Clinton Creek microbialite (ID 4532705.3) and sediment (ID 4532704.3) metagenomes were loaded into STAMPS then compared against arctic mats and sediments: Ward Hunt Ice Shelf mat (ID 4532782.3), MarkHam Ice Shelf (ID 4532781.3), and Lost Hammer Sediments (ID 4532786.3), mats from Yellowstone: Octopus Springs Mat (ID 4443749.3), and Mushroom Springs Mat (ID 4443762.3) and Antarctica mats, marine derived lakes, freshwater lakes, and marine; McMurdo ice shelf mat (ID 4532780.3), Marine Lake 1 (ID 4443683), Marine Lake 5 (ID 4443682.3), Marine 8 (ID 4443686.3), Marine 9 (ID 4443687.3), and Ace Lake (ID 4443684.3) using a multiple group ANOVA in STAMP by SEED subsystems (Level III) annotations by post-hoc tests (Tukey-Kramer at 0.95), an effect size (Eta-squared) and multiple test correction using the Benjamini-Hochberg FDR procedure. Clinton Creek microbialites were further examined against polar mats, Cuatro Ciénegas microbialites (4440060.4, 4440067.3) and marine stromatolites from Highbourne Cay (ID 4440061.3) in STAMPs by a one sided G-test (w/Yates' + Fisher's), alternative to the chi-squared, with asymptomatic confidence intervals (0.95) using Benjamini-Hochberg FDR procedure.

### Metagenomic data depositing

All the data used in this study is freely available and available for public access from the MG-RAST metagenomics analysis server. From MG-RAST, it is listed in the project name Yukon microbialites under the names Clinton Creek microbialite (ID 4532705.3) and sediment contigs (ID 4532704.3).

## Results and discussion

### Non-database based community properties

Based on GC content Clinton Creek the microbialite microbial communities are clearly distinct from the sediment microbial communities. The GC content was higher in the microbialites compared to the sediments; whereas, protein isoelectric points (pI) were similar (Figure S1). The GC content was lower in sediments likely due to the higher presence of low GC containing microbes belonging to phyla such as Bacteroides and Firmicutes. The higher GC content in the microbialite data is likely due to the high relative abundance of sequences assigned to anoxic photoheterotrophic Alphaproteobacteria, including Rhodobacterales (59–65% GC content) and Rhodomicrobium (62.2% GC content). GC content across bacterial genomes can be highly variable amongst microbial taxa, although amino-acid usage is typically similar, which is similar to our data on GC and pI content observed in the metagenomes (Lightfield et al., 2011; Figure S1).

### Community composition

**Figure 2:**
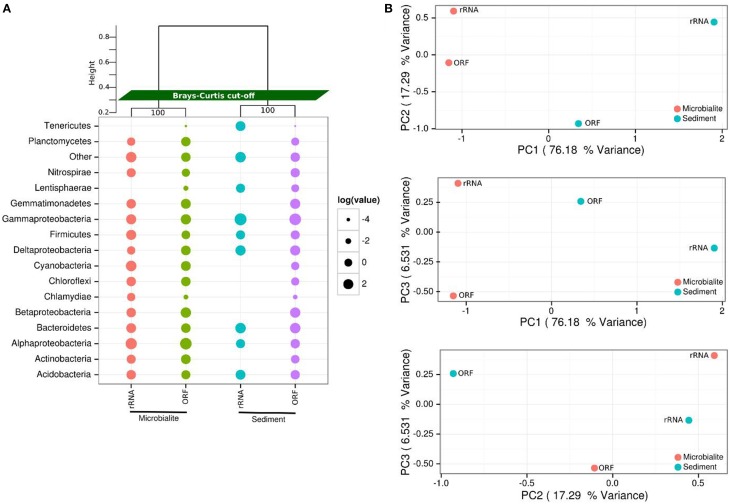
**Microbial community structure of Clinton Creek metagenomes**. **(A)** Dot pot of representative taxonomic groups from Clinton Creek sediments and microbialites using RefSeq (protein coding ORFs) and M5RNA (rRNA, MG-RAST rRNA database) in log relative abundances. Samples were clustered (top) by ward clustering matrix using bootstraping of one thousand replications with Bray-Curtis distance cut-offs. “Other” denotes low abundance taxa that were < 1% of the total ORF or rRNA, individually, but were all combined here into one point. **(B)** PCAs of top 25 taxonomic groups from Clinton Creek sediments and microbialites by RefSeq (ORFs) and M5RNA (rRNA, MG-RAST rRNA database) classification using ward clustering matrix followed by bootstrapping of one thousand replications with Bray-Curtis distance cut-offs.

The microbial communities within Clinton Creek are distinct from each other (Figure 2) and are dominated by differing compositions of Proteobacteria and Cyanobacteria. Proteobacteria comprised >50% of the ORFs and >35% of the 16S sequences recovered from the Clinton Creek sediments and microbialites (Figure 2). The microbialite contigs were dominated by anoxic photoheterotrophic Alphaproteobacteria (e.g., Rhodobacterales) (Figure 2A). In contrast, sediments contigs had greater abundance nitrogen-fixing Gammaproteobacteria (e.g., *Pseudomonas* spp.) (Figure 2A). Alphaproteobacteria are commonly found amongst, and are likely a critical component of, the microbialite-forming microbial consortium due to their role in nitrogen fixation, even in the presence of heterocystous cyanobacteria (Havemann and Foster, 2008). It has been suggested that prior to the evolution of cyanobacteria, anoxic phototrophs like Rhodobacterales could have had a role in the formation of Precambrian stromatolites (Bosak et al., 2007).

Deltaproteobacteria represented ~10% of the predicted Proteobacterial ORFs (based on RefSeq) from the sediments and microbialites (Figure 2A). The microbialite contigs consisted mainly of *Myxococcus* spp. whereas, members of the Desulfuromonadales dominated in the sediments. *Myxococcus* spp. are abundant in a variety of microbialite-forming systems and can mediate precipitate carbonate through the release of ammonium (Jimenez-Lopez et al., 2011). The microbialites and sediments had similar representation from Desulfurovibrionales, Desulfobacterales and Syntrophobacterales, the major orders of dissimilatory sulfate reducers. The dissimilatory sulfate-reducers in the Deltaproteobacteria may be critical drivers of the “the alkalinity engine,” thereby inducing carbonate precipitation (Gallagher et al., 2012). Finding the major dissimilatory sulfate-reducing groups of bacteria (Desulfurovibrionales, Desulfobacterales, and Syntrophobacterales) in Clinton Creek microbialites is not surprising; however, their abundances were similar in the sediments, including genes involved in dissimilatory sulfate-reduction. Thus, the sediments and ground water likely generate alkalinity and could also be the source(s) of these dissimilatory sulfate-reducing bacteria in microbialites. For example, sulfate-reducing bacteria may be transported as spores from other environments and then disperse in microbialite cyanobacterial mats.

Cyanobacteria were the fourth most abundant group, comprising 6.1% of the total contigs in the microbialites (Figure 2A). The microbialites had 4-fold more protein coding ORFs that were classified as cyanobacteria than the sediments (6.1–1.4%, Figure 2A). The cyanobacterial ribosomal sequences were detected in the microbialites only (based on M5RNA database) and from only filamentous cyanobacteria genera, which include Tolypothrix, Leptolyngbya, and unclassified Antarctic cyanobacteria. In contrast, no Cyanobacteria ribosomal sequences (e.g., rDNA) were recovered from the sediments (Figure 2A; M5RNA). No Cyanobacteria ribosomal sequences (e.g., rDNA) were detected in the sediments due to very low abundance sediments. Microbialite contigs based on RefSeq classification had higher abundances of filamentous genera including Microcoleus, Lyngbya, Nodularia, and Anabaena, and more unicellular calcifying Synechococcus, than the sediments. The sediments had fewer filamentous cyanobacteria genera as a whole and fewer unicellular cyanobacteria (e.g., Synechococcus) contigs. Cyanobacteria likely drive microbialite formation in Clinton Creek by increasing carbon biomass in the form of carbon-rich EPS, which supports the growth of the entire heterotrophic microbial consortium through carbon fixation, which in turn contributes to carbonate precipitation by increasing the saturation index (Dupraz and Visscher, 2005; Braissant et al., 2007; Dupraz et al., 2009; McCutcheon et al., 2014).

The phylum Gemmatimonadetes was present in both the sediments and microbialite contigs, and comprised 7–8% of the protein coding ORFs (Figure 2A). To our knowledge, this is the first report of protein sequences from Gemmatimonadetes in microbialites based on metagenomic data, although they were not restricted to that environment. The Gemmatimonadetes contigs annotated mainly as hypothetical proteins; however, positive Gemmatimonadetes annotations included ATPases, Zn-dependent peptidases and glucose/sorbosone dehydrogenase-like genes. Glucose/sorbosone dehydrogenases transform various sugar moieties into vitamins, including L-ascorbic acid (vitamin C), or can make D-glucono-1,5-lactone from D-glucose, which can acidify the extracellular environment, which may lead to dissolution of carbonate by heterotrophic process (Dupraz and Visscher, 2005; Miyazaki et al., 2006; Fender et al., 2012). Although their estimated relative abundance is not high, this could in part be because there are few representative Gemmatimonadetes genomes in databases. Ultimately, whether they are involved in microbialite formation, or are just opportunists, or lead to dissolution, needs to be elucidated.

The microbialite and sediment microbial communities were dominated by bacteria with low abundances of eukaryotes and archaea (Table 3). From RefSeq annotations, < 1% of microbialite and sediment contigs were of archaeal origin (Table 3, RefSeq), and no archaeal ribosomal genes were detected in either the sediment or microbialite contigs (Table 3, M5RNA). Clinton Creek microbialites, similar to Highbourne Cay marine microbialites and the freshwater microbialites from Cuatro Ciénegas, had low abundances (< 1%) of archaea and eukaryotes (Breitbart et al., 2009; Khodadad and Foster, 2012; Mobberley et al., 2013). Eukaryotes were rare, as they make up < 1% of the sediment and microbialite contigs of Clinton Creek (Table 3), although common taxa such as diatoms, dinoflagellates, cryptomonads, chlamydomonadales, and fungi were detected. Diatoms and other protists have been observed by microscopy and detected in the metagenomic data from Clinton Creek, but their contribution to the formation of microbialite structures requires further study (Power et al., 2011a). Diatoms may influence carbonate precipitation through photosynthetic alkalinization (Tesson et al., 2008), akin to processes found in cyanobacteria, and/or through the ammonification of amino acids (Castanier et al., 1999).

**Table 3:** **Domain classification of the microbial community in Clinton Creek**.

Domains^*^	**Microbialite (RefSeq)**	**Microbialite (M5RNA)**	**Sediment (RefSeq)**	**Sediment (M5RNA)**
Bacteria	99.05	73.91	98.22	92.50
Eukaryotes	0.60	25.00	1.16	7.50
Archaea	0.32	0.00	0.45	0.00
Viruses	0.02	0.00	0.13	0.00
Unassigned	0.01	1.09	0.04	0.00

* Based on MG-RAST annotations. RefSeq: Protein coding ORFs. M5RNA: Ribosomal rRNA genes.

Clinton Creek microbialites had very low sequence abundance (< 0.1%) of metazoans including nematoda, cryptomonads, platyhelminthes, microsporidia, cnidaria (e.g., hydra) and arthropods (e.g., insects). Our sequence data supports prior microscopy data that similarly showed low abundances of metazoans (Power et al., 2011a). With such a low metazoan abundance, the destructive impact of grazing on the Clinton Creek microbialites is presumably very low. Phosphorus was undetectable down to the parts per million detection limit in Clinton Creek (data not shown). It has been suggested that limitation of phosphorus affects metazon growth in microbialites (Elser et al., 2005). Metazoan grazing is the “prime suspect” in the global decline of microbialites as they remove cyanobacterial mats, thereby negatively impacting microbialite formation by removing the main carbon source and structural components (Grotzinger, 1990).

## Metabolic potential

**Figure 3:**
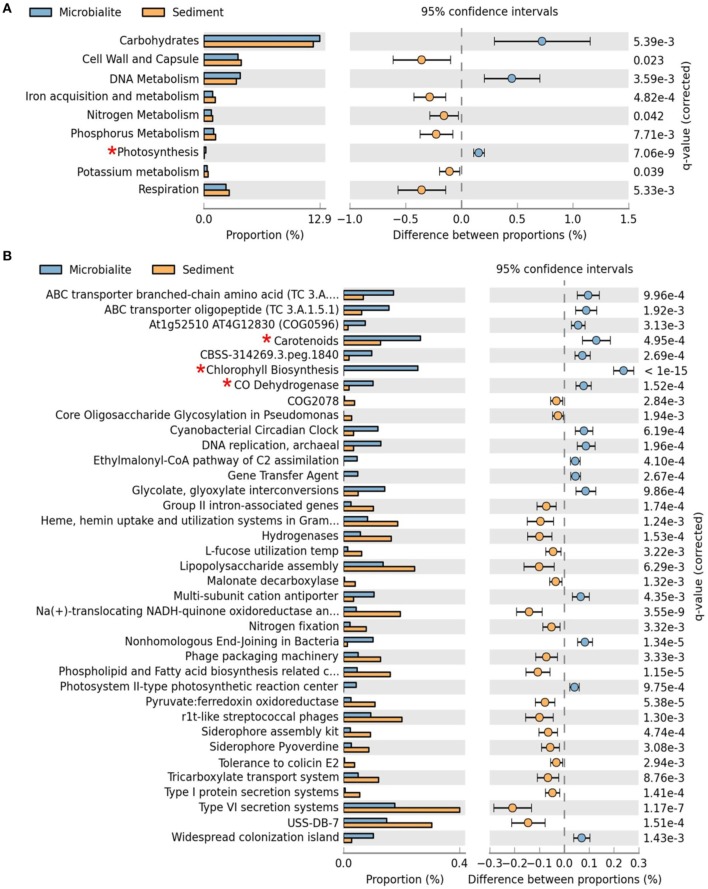
**Extended error plots for functional gene annotations for Clinton Creek metagenomes in STAMP using SEED subsystems. (A)** SEED subsystem level I (highest level classification in SEED). **(B)** SEED subsystem level III (3rd lowest classification in SEED out of four levels). Extended error plots used a one sided G-test (w/Yates' + Fisher's) with asymptomatic confidence intervals (0.95) using Benjamini-Hochberg FDR procedure. ^*^Red asterisks are significant photosynthetic pathways.

**Figure 4:**
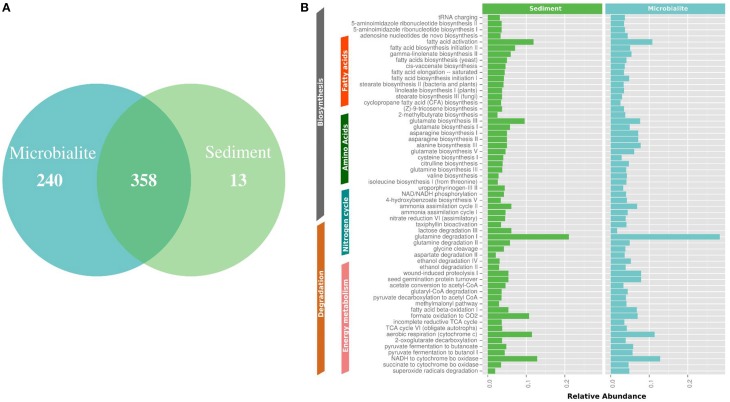
**MetaCyc pathway annotations for Clinton Creek metagenomes. (A)** Venn diagram of MetaCyc pathways. **(B)** The top 40 shared MetaCyc pathways from Venn diagram.

The metabolic potential of the Clinton Creek microbialite metagenome predicts photosynthetic dominance, whereas the sediment metagenomes contained more heterotrophic metabolism (e.g., respiration) (Figure 3A). SEED subsystem level I (i.e., highest functional classification group) annotations indicated that carbohydrate metabolism relating to carbon fixation, DNA metabolism and photosynthesis pathways were significantly more abundant in the microbialites than sediments (Figure 4A, FDR *p* < 0.01). Lower level SEED subsystem predictions (level III) further revealed a higher abundance of photosynthetic pathways (e.g., photosystem II reaction centers and carotenoids and chlorophyll biosynthesis) in microbialites than sediments (Figure 3B, FDR *p* < 0.01). These photosynthetic pathways in microbialites were annotated as filamentous cyanobacteria genera such as Microcoleus, Lyngbya, Nodularia, and Anabaena, which were not found in the sediments.

The metapathway pipeline was used for MetaCyc pathway annotations to complement SEED functional gene annotations. MetaCyc predicted pathways revealed that most pathways were shared between microbialites and sediments (Figure 4A). Only 13 pathways were restricted to the sediments and 240 pathways were identified in the microbialites, while 358 pathways were shared (Figure 4A). The hundred most abundant shared pathways were housekeeping genes with functions such as protein, nucleic acid, lipid, and carbohydrate biosynthesis and degradation (Figure 4B). In the microbialites, MetaCyc annotations predict higher levels of glutamine degradation I, which results in the donation of nitrogen in the form of ammonium, while glutamine biosynthesis appears to be higher in the sediments (Figure 4B). Both the sediments and the microbialites are able to recycle ammonium through ammonium assimilation cycle I-II (Figure 4B). Ammonium donation provides nitrogen, which feeds the primary photosynthetic production of the filamentous cyanobacterial mats in microbialites, which in turn could lead to further carbonate precipitation.

Isotopic analysis of the carbonate minerals composing the microbialite may indicate a dominant process, e.g., alkalinization by phototrophs vs. increased CO_2_ supply via heterotrophic degradation of organic matter. However, microbialites form though complex interactions between the physical and chemical factors with the microbial community. For instance, calcite composing the Pavilion Lake microbialites is enriched in ^13^C by 2.5 ± 0.5‰ relative to calcite that may precipitate in isotopic equilibrium with lake water DIC, (Brady et al., 2009), indicating that alkalinization driven by cyanobacteria. The biomass-associated aragonite within the Clinton Creek microbialites was modestly enriched in ^13^C by 0.8‰ relative to aragonite exhibiting no biomass, which is indicative of carbonate precipitation in association with phototrophs, including cyanobacteria (Power et al., 2011a). Electron microscopy of the microbialites confirmed that phototrophs were associated with carbonate that is enriched in 13C (Power et al., 2011a). A greater proportion of heterotrophic activity within the microbialites may explain why microbialite aragonite was isotopically lighter than periphyton found in the open pit. Omelon et al. (2013) hypothesize that microbialites become progressively lithified as the photosynthetically derived carbonate becomes in-filled through subsequent carbonate precipitation by heterotrophic activity. Similarly, Andres et al. (2006) suggest heterotrophs play a more direct role than phototrophs in the lithification stromatolites from Highborne Cay, Bahamas as indicated by isotopically light aragonite (Andres et al., 2006).

## Comparative metagenomic analysis

**Figure 5:**
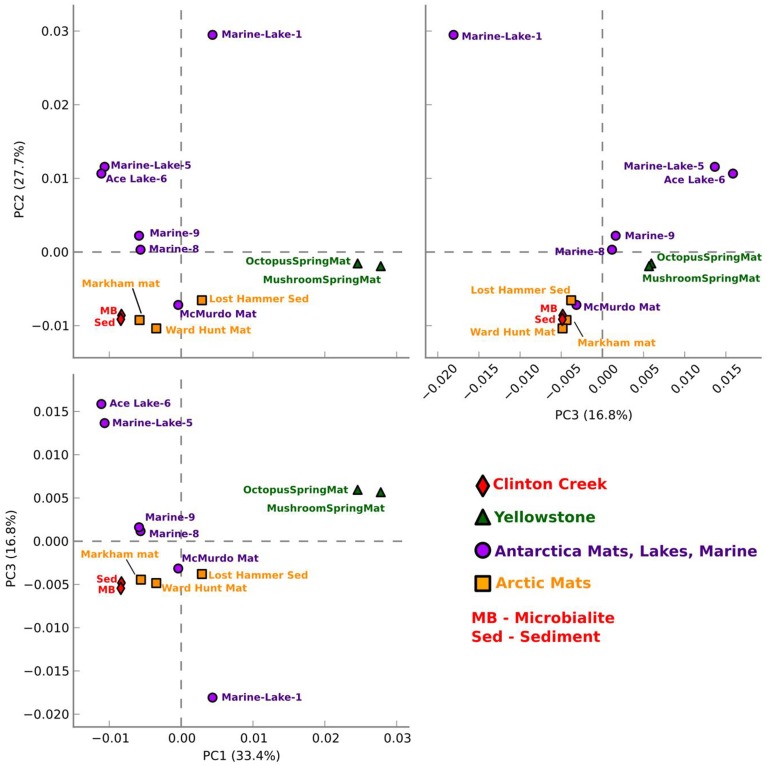
**Functional gene comparative PCA plot for Clinton Creek metagenomes**. Based on ANOVA for multiple groups using SEED subsystem level III in STAMP.

**Figure 6:**
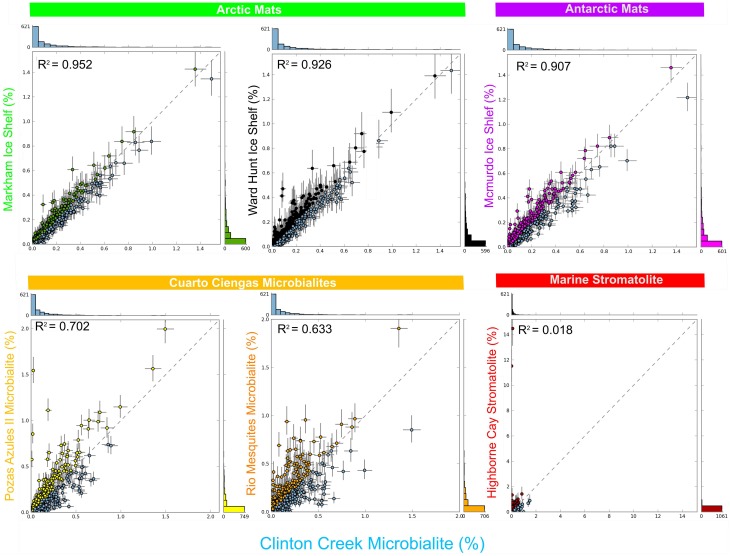
**Scatter plots of functional gene annotations using SEED subsystem level III**. One sided G-test (w/Yates' + Fisher's) with asymptomatic confidence intervals (0.95) using Benjamini-Hochberg FDR procedure in STAMP. Each dot represents a unique functional classification gene.

Clinton Creek microbialite and sediment metagenomes are more functionally related to polar mats than microbialites isolated from marine or tropical ecosystems. Using SEED subsystem level III, PCA indicates better clustering to polar mats and sediments from the Arctic and Antarctica (Figure 5). The Clinton Creek samples cluster most closely Markham Ice shelf and Ward Hunt Ice shelf mats isolated from the Canadian High Arctic (Figure 5; Varin et al., 2012). Markham mats are functionally the most similar based on strong correlation to Clinton Creek microbialites based on SEED subsystem level III (Figure 6, *R*^2^ = 0.952). Overall, polar mats (e.g., Markham, Ward Hunt, and McMurdo) had strong correlation of pathways in SEED than other ecosystems (Figure 6, *R*^2^ > 0.900). Markham mats like Clinton Creek microbialites are dominated by Proteobacteria, and have Gemmatimonadetes present at 3% of the total 16S clones (Bottos et al., 2008). Polar mats, whether on microbialites or on ice shelves, appear to have functional gene similarities; this likely relates to handling shifts in temperature, including temperatures well below freezing (−10°C; Varin et al., 2012).

SEED based functional genes present in Clinton Creek microbialites were also analyzed across both freshwater microbialites from Cuatro Ciénegas and Highbourne Cay stromatolite metagenomes. Clinton Creek microbialites had weak correlations to Pozas Azules II metagenome (Figure 6, *R*^2^ = 0.702), followed by Rio Mesquites (Figure 6, *R*^2^ = 0.633), and Highbourne Cay stromatolite (Figure 6, *R*^2^ = 0.018). Arctic polar mats had stronger correlations for SEED pathways than Cuatro Ciénegas microbialites and Highbourne Cay stromatolite metagenomes. Cuatro Ciénegas microbialites and Highbourne Cay are in tropical climates, which would remove many pathways related to cold-adaptation which are present in Clinton Creek and polar mats (Varin et al., 2012). The Highbourne Cay marine stromatolite had the lowest correlation of SEED functional gene classifications to Clinton Creek microbialites, which further suggests that marine microbialites differ from freshwater microbialites.

Clinton Creek samples were distinct from non-lithifying Octopus and Mushroom spring mats from Yellowstone (Figure 5). These data reveal that Clinton Creek microbialites are closely related to polar mats, due possibly to cold-adaptation, and differ greatly from tropical microbialites. This reveals that under the correct chemistry (e.g., alkaline pH, high DIC, and dissolved Ca^2+^ or Mg^2+^), and with low numbers of metazoans, polar mats on ice shelves could have at least the metabolic potential to make microbialites.

## Clinton creek geochemistry

The key chemical parameters with regard to CaCO_3_ precipitation are pH, and concentrations of Ca^2+^ and DIC. These parameters influence the degree of saturation of a solution as given by the Saturation Index (SI), which is defined as SI = log(IAP/K_sp_), where the IAP is the Ion Activation Product and K_sp_ is the solubility product of a given mineral. Rates of mineral nucleation and precipitation are generally greater with increasing degree of saturation (De Yoreo and Vekilov, 2003). Speciation calculations using PHREEQC (Parkhurst and Appelo, 1999) determined that the average SI for aragonite in the Clinton Creek open pit water is 0.6 vs. 0.72 for calcite of Pavilion Lake (Brady et al., 2009). This may explain the extremely rapid accretion rate in Clinton Creek, which is two orders of magnitude faster than Pavilion Lake microbialites (Brady et al., 2009), and one order faster than Highbourne Cay microbialites (Planavsky and Ginsburg, 2009). Given a similar CaCO_3_ saturation index as Pavilion Lake, the relatively rapid formation of Clinton Creek microbialites cannot be explained by the bulk chemical parameters of the open pit water. Furthermore, the microbialites and surrounding sediments experience nearly the same environmental conditions (e.g., nutrient availability, bulk water chemistry, and lighting). Consequently, we are able to differentiate between the environmental and microbial controls on carbonate precipitation through a comparative analysis of the microbialites vs. the surrounding sediment using metagenomic analysis. The surrounding sediments do contain some aragonite (Power et al., 2011a); however, it is clear that carbonate precipitation rates are much faster in the microbialites given their greater abundance of aragonite. These finding suggest that microbialite formation in Clinton Creek is indeed driven by the local microbial community. Microbial metabolism is expected to significantly modify the water chemistry in the interstitial waters of the microbialites (Dupraz et al., 2009). On a geologic and even a human timescale, Clinton Creek microbialites are exceedingly young, and it may be that their rapid accretion rates will not extend into the future.

Our data suggest that polar mats have the metabolic potential to make microbialites under the correct chemical conditions. Further work is needed to definitively ascertain specific microbe influence in terms of the speed of microbialite formation. In the future, this may provide an avenue for us to engineer microbial communities to store atmospheric carbon through biolithification, especially given the recent, anthropogenic origin of the Clinton Creek site. Biogenic carbonate deposits are the largest reservoirs of carbon on Earth and could provide a cost-efficient method of carbon sequestration for greenhouse gas emissions (Falkowski et al., 2000). Passive carbonation and carbon capture has been documented within the Clinton Creek mine tailings, leading to the proposition that microbially-mediated carbonate precipitation is a means to ameliorate carbon emissions from mining operations (Power et al., 2011a).

## Conclusions

The northernmost microbialites known are located at subarctic Clinton Creek (Yukon, Canada). DNA from representative microbialites was extracted and directly sequenced, without bias from DNA amplification, and used to produce the largest set of assembled metagenomic data from a freshwater microbialite-forming ecosystem. The data revealed a high proportion of photosynthetic genes that were absent in the surrounding sediments, implying that microbialite formation is driven by photosynthesis-induced alkalinization, which is supported by 13C isotopic enrichment (Power et al., 2011b). Predicted metabolic pathways overlapped extensively between microbialite and sediment communities, particularly with respect to housekeeping genes; however, they have distinct core communities with microbialites dominated by Alphaproteobacteria (mainly anoxic phototrophs like Rhodobacterales) and sediments dominated by Gammaproteobacteria (mainly heterotrophic nitrogen-fixing *Pseudomonas* spp.).

While Clinton Creek microbialites shared some functional potential with microbialites from Cuatro Ciénegas, they shared far greater relation to Arctic mats (e.g., Markham and Ward Hunt), possibly due to cold-adaptation facilitated by long winters. The shared metabolic potential between Clinton Creek microbialites and polar mats from ice shelves, suggests that under favorable geochemical conditions, (e.g., alkaline pH, high DIC, and dissolved Ca^2+^ or Mg^2+^), Arctic mats have the metabolic potential to form microbialites.

This study illustrates that cyanobacteria generate alkalinity and support heterotrophic communities, which have the potential to drive the formation of microbialites at Clinton Creek. Together, this suggests that an anthropogenic environment can foster microbial communities capable of mediating carbonate precipitation, and that these microbes could offer an effective means of carbon sequestration (Power et al., 2011a,b). Microbially-mediated carbonate precipitation is an environmentally safe and novel process that could be harnessed to provide a cost-efficient strategy for the long-term storage of anthropogenic greenhouse gasses (e.g., CO_2_).

### Conflict of interest statement

The authors declare that the research was conducted in the absence of any commercial or financial relationships that could be construed as a potential conflict of interest.

## References

[B1] Allen M. A. Goh F. Burns B. P. Neilan B. A. (2009). Bacterial, archaeal and eukaryotic diversity of smooth and pustular microbial mat communities in the hypersaline lagoon of Shark Bay. Geobiology 7, 82–96. 10.1111/j.1472-4669.2008.00187.x 19200148

[B2] Andres M. S. Sumner D. Y. Reid R. P. Swart P. K. (2006). Isotopic fingerprints of microbial respiration in aragonite from Bahamian stromatolites. Geology 34, 973–976. 10.1130/G22859A.1

[B3] Arp G. Reimer A. Reitner J. (2001). Photosynthesis-induced biofilm calcification and calcium concentrations in Phanerozoic oceans. Science 292, 1701–1704. 10.1126/science.1057204 11387471

[B4] Boisvert S. Laviolette F. Corbeil J. (2010). Ray:simultaneous assembly of reads from a mix of high-throughput sequencing technologies. J. Comput. Biol. 11, 1519–1533. 10.1089/cmb.2009.0238 20958248 PMC3119603

[B5] Boisvert S. Raymond F. Godzaridis E. Laviolette F. Corbeil J. (2012). Ray Meta: scalable de novo metagenome assembly and profiling. Genome Biol. 13:R122. 10.1186/gb-2012-13-12-r122 23259615 PMC4056372

[B6] Bosak T. Greene S. E. Newman D. K. (2007). A likely role for anoxygenic photosynthetic microbes in the formation of ancient stromatolites. Proc. Natl. Acad. Sci. U.S.A. 5, 119–126. 10.1111/j.1472-4669.2007.00104.x 20890383 PMC2947360

[B7] Bosak T. Liang B. Wu T. D. Templer S. P. Evans A. Vali H.. (2012). Cyanobacterial diversity and activity in modern conical microbialites. Geobiology 10, 384–401. 10.1111/j.1472-4669.2012.00334.x 22713108

[B8] Bottos E. M. Vincent W. F. Greer C. W. Whyte L. G. (2008). Prokaryotic diversity of arctic ice shelf microbial mats. Environ. Microbiol. 10, 950–966. 10.1111/j.1462-2920.2007.01516.x 18215157

[B9] Brady A. L. Slater G. Laval B. Lim D. S. (2009). Constraining carbon sources and growth rates of freshwater microbialites in Pavilion Lake using ^14^C analysis. Geobiology 7, 544–555. 10.1111/j.1472-4669.2009.00215.x 19702837

[B10] Braissant O. Decho A. W. Dupraz C. Glunk C. Przekop K. M. Visscher P. T. (2007). Exopolymeric substances of sulfate-reducing bacteria: interactions with calcium at alkaline pH and implication for formation of carbonate minerals. Geobiology 5, 401–411. 10.1111/j.1472-4669.2007.00117.x

[B11] Breitbart M. Hoare A. Nitti A. Siefert J. Haynes M. Dinsdale E.. (2009). Metagenomic and stable isotopic analyses of modern freshwater microbialites in Cuatro Ciénegas, Mexico. Environ. Microbiol. 11, 16–34. 10.1111/j.1462-2920.2008.01725.x 18764874

[B12] Burne R. V. Moore L. S. (1987). Microbialites: organosedimentary deposits of benthic microbial communities. Palaios 2, 241–254. 10.2307/3514674

[B13] Burns B. P. Goh F. Allen M. Neilan B. A. (2004). Microbial diversity of extant stromatolites in the hypersaline marine environment of Shark Bay, Australia. Environ. Microbiol. 6, 1096–1101. 10.1111/j.1462-2920.2004.00651.x 15344935

[B14] Castanier S. Le Metayer-Levrel G. Perthuisot J. P. (1999). Ca-carbonates precipitation and limestone genesis the microbiogeologist point of view. Sediment. Geol. 126, 9–23. 10.1016/S0037-0738(99)00028-7

[B15] Centeno C. M. Legendre P. Beltran Y. Alcantara-Hernandez R. J. Lidstrom U. E. Ashby M. N.. (2012). Microbialite genetic diversity and composition related to environmental variables. FEMS Microbiol. Ecol. 82, 724–735. 10.1111/j.1574-6941.2012.01447.x 22775797

[B16] Chivas A. R. Torgersen T. Polach H. A. (1990). Growth rates and Holocene development of stromatolites from Shark Bay, Western Australia. Aust. J. Earth Sci. 37, 113–121. 10.1080/08120099008727913

[B17] Couradeau E. Benzerara K. Moreira D. Gérard E. Kaźmierczak J. Tavera R.. (2011). Prokaryotic and eukaryotic community structure in field and cultured microbialites from the alkaline lake Alchichica (Mexico). PLoS ONE 6:e28767. 10.1371/journal.pone.0028767 22194908 PMC3237500

[B18] De Yoreo J. J. Vekilov P. G. (2003). Principles of crystal nucleation and growth, in Biomineralization, eds Dove P. M. Deyoreo J. J. Weiner S. (Washington, WA: Mineralogical Soc America), 57–93.

[B19] Dupraz C. Reid R. P. Braissant O. Decho A. W. Norman R. S. Visscher P. T. (2009). Processes of carbonate precipitation in modern microbial mats. Earth Sci. Rev. 96, 141–162. 10.1016/j.earscirev.2008.10.005

[B20] Dupraz C. Visscher P. T. (2005). Microbial lithification in marine stromatolites and hypersaline mats. Trends Microbiol. 13, 429–438 10.1016/j.tim.2005.07.008 16087339

[B21] Elser J. J. Schampel J. H. Kyle M. Watts J. Carson E. W. Dowling T. E. (2005). Response of grazing snails to phosphorus enrichment of modern stromatolitic microbial communities. Freshw. Biol. 50, 1826–1835. 10.1111/j.1365-2427.2005.01453.x

[B22] Falkowski P. Scholes R. J. Boyle E. Canadell J. Canfield D. Elser J.. (2000). The global carbon cycle: a test of our knowledge of earth as a system. Science 290, 291–296. 10.1126/science.290.5490.291 11030643

[B23] Fender J. E. Bender C. M. Stella N. A. Lahr R. M. Kalivoda E. J. Shanks R. M. (2012). *Serratia marcescens* quinoprotein glucose dehydrogenase activity mediates medium acidification and inhibition of prodigiosin production by glucose. Appl. Environ. Microbiol. 78, 6225–6235 10.1128/aem.01778-12 22752173 PMC3416624

[B24] Ferris F. G. Thompson J. B. Beveridge T. J. (1997). Modern freshwater microbialites from Kelly Lake, British Columbia, Canada. Palaios 12, 213–219. 10.2307/3515423

[B25] Gallagher K. L. Kading T. J. Braissant O. Dupraz C. Visscher P. T. (2012). Inside the alkalinity engine: the role of electron donors in the organomineralization potential of sulfate-reducing bacteria. Geobiology 10, 518–530. 10.1111/j.1472-4669.2012.00342.x 22925453

[B26] Gischler E. Gibson M. A. Oschmann W. (2008). Giant Holocene freshwater microbialites, Laguna Bacalar, Quintana Roo, Mexico. Sedimentology 55, 1293–1309. 10.1111/j.1365-3091.2007.00946.x

[B27] Goh F. Allen M. A. Leuko S. Kawaguchi T. Decho A. W. Burns B. P.. (2009). Determining the specific microbial populations and their spatial distribution within the stromatolite ecosystem of Shark Bay. ISME J. 3, 383–396. 10.1038/ismej.2008.114 19092864

[B28] Grotzinger J. P. (1990). Geochemical model for proterozoic stromatolite decline. Am. J. Sci. 290, 80–103.

[B29] Grotzinger J. P. Knoll A. H. (1999). Stromatolites in Precambrian carbonates: evolutionary mileposts or environmental dipsticks? Ann. Rev. Earth Planet. Sci. 27, 313–358. 10.1146/annurev.earth.27.1.313 11543060

[B30] Havemann S. A. Foster J. S. (2008). Comparative characterization of the microbial diversities of an artificial microbialite model and a natural stromatolite. Appl. Environ. Microbiol. 74, 7410–7421. 10.1128/AEM.01710-08 18836014 PMC2592906

[B31] Htoon M. (1979). Geology of the Clinton Creek asbestos deposit, Yukon Territory, in Department of Earth and Ocean Sciences (Vancouver: University of British Columbia). Available online at: http://circle.ubc.ca/handle/2429/21374

[B32] Jimenez-Lopez C. Chekroun K. B. Jroundi F. Rodríguez-Gallego M. Arias J. M. González-Muñoz M. T. (2011). *Myxococcus xanthus* colony calcification: A study to better understand the processes involved in the formation of this stromatolite-like structure. Adv. Strom. Geobiol. 131, 161–181. 10.1007/978-3-642-10415-2_11

[B33] Khodadad C. L. Foster J. S. (2012). Metagenomic and metabolic profiling of nonlithifying and lithifying stromatolitic mats of Highborne Cay, The Bahamas. PLoS ONE 7:e38229. 10.1371/journal.pone.0038229 22662280 PMC3360630

[B34] Kiełbasa S. M. Wan R. Sato K. Horton P. Frith M. C. (2011). Adaptive seeds tame genomic sequence comparison. Genome Res. 3, 487–493. 10.1101/gr.113985.110 21209072 PMC3044862

[B35] Konwar K. M. Hanson N. W. Pagé A. P. Hallam S. J. (2013). MetaPathways: a modular pipeline for constructing pathway/genome databases from environmental sequence information. BMC Bioinform. 14:202. 10.1186/1471-2105-14-202 23800136 PMC3695837

[B36] Laval B. Cady S. L. Pollack J. C. McKay C. P. Bird J. S. Grotzinger J. P.. (2000). Modern freshwater microbialite analogues for ancient dendritic reef structures. Nature 407, 626–629. 10.1038/35036579 11034210

[B37] Lightfield J. Fram N. R. Ely B. (2011). Across bacterial phyla, distantly-related genomes with similar genomic GC content have similar patterns of amino acid usage. PLoS ONE 6:e17677. 10.1371/journal.pone.0017677 21423704 PMC3053387

[B38] Lim D. S. S. Laval B. E. Slater G. Antoniades D. Forrest A. L. Pike W. (2009). Limnology of Pavilion Lake, B.C., Canada—Characterization of a microbialite forming environment. Fundam. Appl. Limnol. 173, 329–351. 10.1127/1863-9135/2009/0173-0329

[B39] López-García P. Kazmierczak J. Benzerara K. Kempe S. Guyot F. Moreira D. (2005). Bacterial diversity and carbonate precipitation in the giant microbialites from the highly alkaline Lake Van, Turkey. Extremophiles 9, 263–274. 10.1007/s00792-005-0457-0 15959626

[B40] Ludwig R. Al-Horani F. A. de Beer D. Jonkers H. M. (2005). Photosynthesis-controlled calcification in a hypersaline microbial mat. Limnol. Oceanogr. 50, 1836–1843. 10.4319/lo.2005.50.6.1836

[B41] Lundberg J. McFarlane D. A. (2011). Subaerial freshwater phosphatic stromatolites in Deer Cave, Sarawak—A unique geobiological cave formation. Geomorphology 128, 57–72. 10.1016/j.geomorph.2010.12.022

[B42] Maliva G. R. Missimer M. T. Leo C. K. Statom A. R. Dupraz C. Lynn M. (2000). Unusual calcite stromatolites and pisoids from a landfill leachate collection system. Geology 28, 931–934. 10.1130/0091-7613(2000)28<931:UCSAPF>2.0.CO;2

[B43] McCutcheon J. Power I. M. Harrison A. L. Dipple G. M. Southam G. (2014). A greenhouse-scale photosynthetic microbial bioreactor for carbon sequestration in magnesium carbonate minerals. Environ. Sci. Technol. 48, 9142–9151. 10.1021/es500344s 25072950

[B44] Meyer F. D. Paarmann M. D'souza R. Olson E. M. Glass M. Kubal T.. (2008). The Metagenomics RAST server - A public resource for the automatic phylogenetic and functional analysis of metagenomes. BMC Bioinformatics 9:386. 10.1186/1471-2105-9-386 18803844 PMC2563014

[B45] Miyazaki T. Sugisawa T. Hoshino T. (2006). Pyrroloquinoline quinone-dependent dehydrogenases from Ketogulonicigenium vulgare catalyze the direct conversion of L-sorbosone to L-ascorbic acid. Appl. Environ. Microbiol. 72, 1487–1495. 10.1128/AEM.72.2.1487-1495.2006 16461703 PMC1392885

[B46] Mobberley J. M. Khodadad C. L. Foster J. S. (2013). Metabolic potential of lithifying cyanobacteria-dominated thrombolitic mats. Photosyn. Res. 118, 125–140. 10.1007/s11120-013-9890-6 23868401 PMC5766932

[B47] Omelon C. R. Brady A. L. Slater G. F. Laval B. Lim D. S. S. Southam G. (2013). Microstructure variability in freshwater microbialites, Pavilion Lake, Canada. Paleogeogr. Paleoclimatol. Paleoecol. 392, 62–70. 10.1016/j.palaeo.2013.08.017

[B48] Papineau D. Walker J. J. Mojzsis S. J. Pace N. R. (2005). Composition and structure of microbial communities from stromatolites of Hamelin Pool in Shark Bay, Western Australia. Appl. Environ. Microbiol. 71, 4822–4832. 10.1128/AEM.71.8.4822-4832.2005 16085880 PMC1183352

[B49] Parkhurst D. L. Appelo C. A. J. (1999). User's Guide to PHREEQC (version 2) A Computer Program for Speciation, Batch Reaction, One-dimensional Transport, and Inverse Geochemical Calculations: U.S., Geological Survey Water-Resources Investigations Report, 99–4259.

[B50] Parks D. H. Beiko R. G. (2010). Identifying biologically relevant differences between metagenomic communities. Bioinformatics 26, 715–721. 10.1093/bioinformatics/btq041 20130030

[B51] Perry R. S. Mcloughlin N. Lynne B. Y. Sephton M. A. Oliver J. D. Perry C. C. (2007). Defining biominerals and organominerals: direct and indirect indicators of life. Sed. Geol. 201, 157–179. 10.1016/j.sedgeo.2007.05.014

[B52] Planavsky N. Ginsburg R. N. (2009). Taphonomy of modern marine Bahamian microbialites. Palaios 24, 5–17. 10.2110/palo.2008.p08-001r

[B53] Power I. M. Wilson S. A. Dipple G. M. Southam G. (2011a). Modern carbonate microbialites from an asbestos open pit pond, Yukon, Canada. Geobiology 9, 180–195. 10.1111/j.1472-4669.2010.00265.x 21231993

[B54] Power I. M. Wilson S. A. Small D. P. Dipple G. M. Wan W. Southam G. (2011b). Microbially mediated mineral carbonation: roles of phototrophy and heterotrophy. Environ. Sci. Technol. 45, 9061–9068. 10.1021/es201648g 21879741

[B55] Reid R. P. Visscher P. T. Decho A. W. Stolz J. F. Bebout B. M. Dupraz C.. (2000). The role of microbes in accretion, lamination and early lithification of modern marine stromatolites. Nature 406, 989–999. 10.1038/35023158 10984051

[B56] Rho M. Tang H. Ye Y. (2010). FragGeneScan: predicting genes in short and error-prone reads. Nucleic Acids Res. 38, e191. 10.1093/nar/gkq747 20805240 PMC2978382

[B57] Riding R. (2011). Microbialites, Stromatolites, and Thrombolites, in Encyclopedia of Geobiology, Encyclopedia of Earth Sciences Series, eds Reitner J. Thiel V. (Heidelberg: Springer), 635–654.

[B58] Russell J. A. Brady A. L. Cardman Z. Slater G. F. Lim D. S. S. Biddle J. F. (2014). Prokaryote populations of extant microbialites along a depth gradient in Pavilion Lake, British Columbia, Canada. Geobiology 3, 250–264. 10.1111/gbi.12082 24636451

[B59] Santos F. Peña A. Nogales B. Soria-Soria E. del Cura M. A. González-Martín J. A.. (2010). Bacterial diversity in dry modern freshwater stromatolites from Ruidera Pools Natural Park, Spain. Syst. Appl. Microbiol. 33, 209–221. 10.1016/j.syapm.2010.02.006 20409657

[B60] Schopf J. W. (2006). Fossil evidence of Archean life. Phil. Trans. R. Soc. B. 361, 869–885. 10.1098/rstb.2006.1834 16754604 PMC1578735

[B61] Schultze-Lam S. Fortin D. Davis B. S. Beveridge T. J. (1996). Mineralization of bacterial surfaces. Chem. Geol., 132, 171–181. 10.1016/S0009-2541(96)00053-8

[B62] Slaughter M. Hill R. J. (1991).The influence of organic matter in organogenic dolomitization. J. Sediment. Petrol. 61, 296–303. 10.1306/D42676F9-2B26-11D7-8648000102C1865D

[B63] Spanos N. Koutsoukos P. G. (1998). Kinetics of precipitation of calcium carbonate in alkaline pH at constant supersaturation. Spontaneous and seeded growth. J. Phys. Chem. B 102, 6679–6684. 10.1021/jp981171h

[B64] Suzuki R. Shimodaira H. (2006). Pvclust: an R package for assessing the uncertainty in hierarchical clustering. Bioinformatics 22, 1540–1542. 10.1093/bioinformatics/btl117 16595560

[B65] Tesson B. Gaillard C. Martin-Jézéquel V. (2008). Brucite formation mediated by the diatom Phaeodactylum tricornutum. Mar. Chem. 109, 60–76. 10.1016/j.marchem.2007.12.005

[B66] Thompson J. B. Ferris F. G. (1990). Cyanobacterial precipitation of gypsum, calcite, and magnesite from natural alkaline lake water. Geology 18, 995–998.

[B67] Van Lith Y. Vasconcelos C. Warthmann R. McKenzie J. A. (2003).Microbial fossilization in carbonate sediments; a result of thebacterial surface involvement in carbonate precipitation. Sedimentology 50, 237–245. 10.1046/j.1365-3091.2003.00550.x

[B68] Varin T. Lovejoy C. Jungblut A. D. Vincent W. F. Corbeil J. (2012). Metagenomic analysis of stress genes in microbial mat communities from Antarctica and the High Arctic. Appl. Environ. Microbiol. 78, 549–559. 10.1128/AEM.06354-11 22081564 PMC3255749

[B69] Von Knorre H. Krumbein W. E. (2000). Bacterial calcification, in Microbial Sediments, eds Riding R. E. Awramik S.M. (Berlin: Springer), 25–31.

[B70] White R. A. III. Blainey P. C. Fan H. C. Quake S. R. (2009). Digital PCR provides sensitive and absolute calibration for high throughput sequencing. BMC Genomics 10:116. 10.1186/1471-2164-10-116 19298667 PMC2667538

[B71] White R. A. III. Grassa C. J. Suttle C. A. (2013a). First draft genome sequence from a member of the genus *Agrococcus*, isolated from modern microbialites. Genome Announc. 1, e00391–e00313. 10.1128/genomeA.00391-13 23814108 PMC3695436

[B72] White R. A. III. Grassa C. J. Suttle C. A. (2013b). Draft genome sequence of *Exiguobacterium pavilionensis* strain RW-2, with wide thermal, salinity, and pH tolerance, isolated from modern freshwater microbialites. Genome Announc. 1, e00597–e00513. 10.1128/genomeA.00597-13 23929485 PMC3738901

[B73] White R. A. III. Suttle C. A. (2013). The Draft Genome Sequence of *Sphingomonas paucimobilis* Strain HER1398 (Proteobacteria), Host to the Giant PAU Phage, indicates that it is a member of the genus *Sphingobacterium* (Bacteroidetes). Genome Announc. 1, e00598–e00513. 10.1128/genomeA.00598-13 23929486 PMC3738902

[B74] Wickham H. (2009). ggplot2: Elegant Graphics for Data Analysis. New York, NY: Springer.

[B75] Wilson S. A. Dipple G. M. Power I. M. Thom J. M. Anderson R. G. Raudsepp M. (2009). Carbon dioxide fixation within mine wastes of ultramafic-hosted ore deposits: examples from the Clinton Creek and Cassiar chrysotile deposits, Canada. Econ. Geol. 104, 95–112. 10.2113/gsecongeo.104.1.95

[B76] Wu S. Zhu Y. (2012). ProPAS: standalone software to analyze protein properties. Bioinformation 8, 167–169. 10.6026/97320630008167 22368391 PMC3283891

