# Supplementary material for: Metagenomic analysis reveals that modern microbialites and polar microbial mats have similar taxonomic and functional potential
Source: Front Microbiol. 2015 Sep 23;6:966. doi: 10.3389/fmicb.2015.00966 (PMC4585152; doi:10.3389/fmicb.2015.00966)
Supplement: Supplementary file 1 [file DataSheet1.PDF]

Figure S1

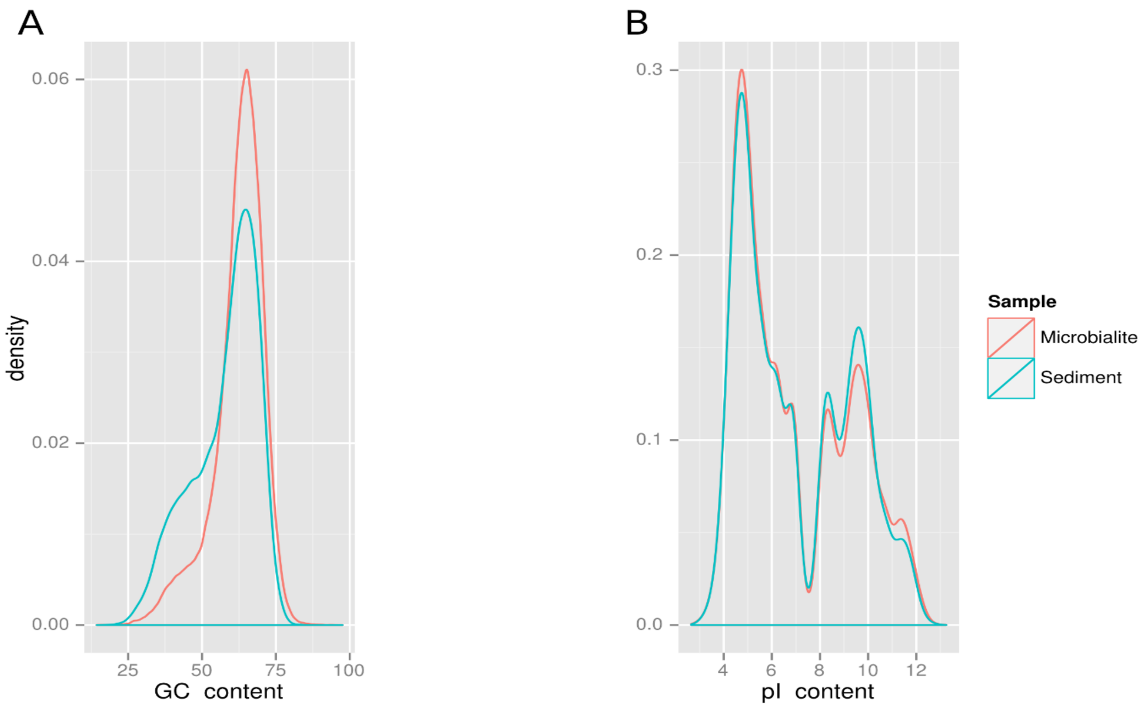

Suppl. Figure 1 | Molecular properties of the Clinton Creek microbialite and sediment contigs. A) GC content (%) B) Predicted protein isoelectric (pI) content.
